# Direct Proteolytic Cleavage of NLRP1B Is Necessary and Sufficient for Inflammasome Activation by Anthrax Lethal Factor

**DOI:** 10.1371/journal.ppat.1003452

**Published:** 2013-06-20

**Authors:** Joseph Chavarría-Smith, Russell E. Vance

**Affiliations:** Division of Immunology & Pathogenesis, Department of Molecular & Cell Biology and Cancer Research Laboratory, University of California, Berkeley, Berkeley, California, United States of America; University of California Los Angeles, United States of America

## Abstract

Inflammasomes are multimeric protein complexes that respond to infection by recruitment and activation of the Caspase-1 (CASP1) protease. Activated CASP1 initiates immune defense by processing inflammatory cytokines and by causing a rapid and lytic cell death called pyroptosis. Inflammasome formation is orchestrated by members of the nucleotide-binding domain and leucine-rich repeat (NLR) or AIM2-like receptor (ALR) protein families. Certain NLRs and ALRs have been shown to function as direct receptors for specific microbial ligands, such as flagellin or DNA, but the molecular mechanism responsible for activation of most NLRs is still poorly understood. Here we determine the mechanism of activation of the NLRP1B inflammasome in mice. NLRP1B, and its ortholog in rats, is activated by the lethal factor (LF) protease that is a key virulence factor secreted by *Bacillus anthracis*, the causative agent of anthrax. LF was recently shown to cleave mouse and rat NLRP1 directly. However, it is unclear if cleavage is sufficient for NLRP1 activation. Indeed, other LF-induced cellular events have been suggested to play a role in NLRP1B activation. Surprisingly, we show that direct cleavage of NLRP1B is sufficient to induce inflammasome activation in the absence of LF. Our results therefore rule out the need for other LF-dependent cellular effects in activation of NLRP1B. We therefore propose that NLRP1 functions primarily as a sensor of protease activity and thus could conceivably detect a broader spectrum of pathogens than just *B. anthracis*. By adding proteolytic cleavage to the previously established ligand-receptor mechanism of NLR activation, our results illustrate the remarkable flexibility with which the NLR architecture can be deployed for the purpose of pathogen-detection and host defense.

## Author Summary

Recognition of pathogens by the innate immune system is necessary for initiating an appropriate immune response. The innate immune system must distinguish pathogens from abundant harmless microbes present within the host and the environment, and scale the response appropriately. It has been proposed that the host can respond specifically to pathogens by monitoring common virulence-associated activities, previously termed “patterns of pathogenesis,” that are used by pathogens to survive and replicate within their hosts. For example, pathogens can manipulate host functions by delivering toxins into host cells. In response, the host encodes dedicated cytosolic sensors to detect these toxins, but the molecular basis for how the sensors recognize the toxins is poorly understood. Here we define the molecular mechanism by which a mouse sensor, NLRP1B, directly recognizes the activity of a bacterial toxin, lethal factor. Lethal factor is a protease secreted by *Bacillus anthracis*, the causative agent of anthrax. We show that anthrax lethal factor cleaves NLRP1B and this cleavage event is both necessary and sufficient for the activation of this sensor. Our findings raise the possibility that NLRP1B could sense the activity of other proteases encoded by diverse pathogens.

## Introduction

Recognition of pathogens is an essential first step in the initiation of protective host immune responses. Recognition of pathogens has been shown to be mediated by several families of germ-line encoded receptors that include the Toll-like receptors (TLRs), Nucleotide-binding domain and Leucine-rich Repeat containing proteins (NLRs), and RIG-I-like receptors (RLRs) [1]. Most TLRs, NLRs, and RLRs for which activation mechanisms have been defined appear to function as “pattern recognition receptors” [2] that directly bind to molecular structures called pathogen-associated molecular patterns (PAMPs) that are broadly conserved among many microbes. In addition to detection of PAMPs, it has been previously proposed that the innate immune system might also respond to ‘Patterns of Pathogenesis’, the virulence-associated activities that pathogens utilize to invade or manipulate their hosts [3]. Detection of pathogen-associated activities might be a beneficial innate immune strategy, complementary to PAMP recognition, as it could allow the innate immune system to discriminate pathogenic from non-pathogenic microbes, and scale responses appropriately, despite the fact that pathogenic and non-pathogenic microbes often share the same PAMPs. However, few instances of a molecular mechanism by which a pathogen-encoded activity could be detected have been described in mammals. For example, a previous study showed how pathogen-induced inhibition of protein synthesis by *Legionella pneumophila* could be detected, leading to a specific cytokine response [4], [5]. Disruption of the actin cytoskeletal signaling by bacterial toxins was also found to lead to a protective innate immune response [6], [7] Overall, however, there is still considerable uncertainty as to whether or how ‘patterns of pathogenesis’ are sensed by the innate immune system.

Anthrax lethal toxin (LeTx) is a critical virulence factor secreted by *Bacillus anthracis*. LeTx is composed of two proteins: protective antigen (PA) and lethal factor (LF). PA binds to anthrax toxin receptors on host cells, and subsequently translocates the zinc-metalloprotease, LF, into the cytosol. The canonical proteolytic substrates of LF are mitogen-activated protein kinase kinases (MAPKKs) 1–4 and 6–7 [8], [9]. Cleavage by LF inactivates MAPKKs and results in the disruption of signaling pathways involved in host defense [10], [11]. Macrophages from certain strains of mice and rats respond to LeTx by undergoing a rapid and lytic form of Caspase-1 (CASP1)-dependent cell death called pyroptosis [12]–[16]. The ability to undergo pyroptosis in response to LeTx was genetically mapped to the *Nlrp1b* gene in mice [13], and subsequently to the orthologous *Nlrp1* gene in rats [16]. Importantly, mice harboring an allele of *Nlrp1b* that is responsive to LeTx are protected from challenge with *B. anthracis* spores [17], [18]. This protection correlates with enhanced production of IL-1β, recruitment of neutrophils to the site infection, and decreased bacterial counts, and these processes depend on expression of the interleukin-1 receptor [17], [18]. Despite the importance of NLRP1B in host defense against *B. anthracis*, the mechanism of NLRP1B activation by LF remains unclear.

NLRP1B belongs to the NLR family of innate immune sensors [19]–[21]. Several NLRs, including NLRP1, have been found to assemble into oligomeric complexes, called ‘inflammasomes’ [22], in response to a variety of infectious or noxious stimuli [21]. The primary function of inflammasomes appears to be to form a platform for activation of inflammatory caspase proteases, most notably CASP1, but the molecular mechanism by which NLRs are activated is poorly understood [21]. Although NLRP1 proteins contain NBD and LRR domains, as do all other NLRs, the domain organization of NLRP1 differs from other NLRs in two respects. First, NLRP1 proteins contain a C-terminal Caspase Activation and Recruitment Domain (CARD), whereas the CARDs in other NLRs are usually N-terminal. The second unique feature of NLRP1 proteins is that they contain an unusual domain called the ‘function-to-find’ (FIIND) domain [19]. The FIIND is located between the LRRs and the C-terminal CARD, and was recently shown to undergo an auto-proteolytic processing event that results in the C-terminal CARD being cleaved from the rest of the NLRP1 protein [23]. It is believed that the N- and C-terminal auto-processed fragments of mature NLRP1B remain associated with each other despite cleavage of the polypeptide chain [24]. The FIIND auto-processing event occurs constitutively, prior to NLRP1B activation by LF, but for reasons that remain unclear, is required for the ability of NLRP1 to activate CASP1 [24], [25].

Several inflammasomes have been suggested to be activated upon direct binding to specific bacterial ligands. For example, another NLR-family member, NAIP5, assembles into an inflammasome upon binding to flagellin, whereas the related NAIP2 inflammasome assembles upon binding to the inner rod proteins from a variety of bacterial type III secretion systems [26], [27]. A direct receptor-ligand model also applies to the ALR-family AIM2 inflammasome, which is activated upon direct binding to microbial DNA [28]–[31]. In contrast, certain inflammasomes, notably the NLRP3 inflammasome, are believed not to bind directly to bacterial ligands, but have instead been proposed to respond to virally encoded ion channels [32], bacterial toxins, or other cellular stresses, via indirect mechanisms [21]. However, the molecular basis for how these stresses are sensed by NLRP3 remains unclear. By contrast, the molecular basis for indirect pathogen recognition by plant NLRs has been well-established [33], [34]. For example, the plant NLR RPS2 has been shown to be maintained in an inactive state by its association with RIN4, a host protein that is targeted for degradation by a bacterial protease [35], [36]. RPS2 thus detects the activity of a bacterial protease indirectly by monitoring or ‘guarding’ the integrity of the protease substrate. Direct proteolytic cleavage of a plant NLR by a pathogen-encoded protease has not been described.

NLRP1B responds to the protease activity of LF, as catalytically inactive forms of LF do not activate NLRP1 [37], [38]. This suggests that NLRP1 does not recognize LF via simple receptor-ligand binding, such as that occurs with the NAIP or AIM2 inflammasomes. Boyden and Dietrich initially hypothesized that LF could cleave and activate NLRP1B [13], but evidence for this simple model of NLRP1B activation was not provided. In fact, several groups have demonstrated that the activity of the proteasome is specifically required for this inflammasome and not for other inflammasomes such as the NAIP5/NLRC4 inflammasome [37], [39]. In addition, inhibitors of the N-end rule degradation pathway block NLRP1B activation but do not affect the ability of LF to cleave MAPKKs [40]. In contrast to a model in which NLRP1B is activated upon cleavage by LF, these observations suggest that LF might activate NLRP1B by cleaving and destabilizing a negative regulator of NLRP1B. This ‘indirect’ model resembles the activation of the certain NLRs, e.g., RPS2, in plants. Recently, however, it was shown that the NLRP1 proteins from Fischer rats and BALB/c mice can be directly cleaved near their N-termini by LF [41], [42]. Mutation of the cleavage site in rat NLRP1 rendered NLRP1 resistant to cleavage by LF and also prevented NLRP1 activation in response to LF. These results suggest that cleavage of rat NLRP1 by LF is essential for NLRP1 activation, but it is difficult to rule out the possibility that mutation of the cleavage site disrupted the fold of NLRP1, or rendered NLRP1 non-functional for other reasons. In addition, the site at which LF cleaves rat NLRP1 is not conserved in the mouse [42], and moreover, the functional effects of mutating the mouse cleavage site have not been assessed. Lastly, and most importantly, existing studies have not ruled out the involvement of other LF-dependent cellular events in NLRP1B activation, as cleavage of NLRP1 was not shown to be sufficient for its activation.

Here, we present data that suggest that murine NLRP1B requires LF-dependent cleavage for its activation. We further demonstrate that cleavage is sufficient for NLRP1B inflammasome activation in the absence of LF, which rules out a requirement for cleavage of other LF substrates in activation of NLRP1B. Our results provide evidence for a simple direct mechanism by which an innate immune sensor detects a pathogen-encoded activity. In addition, our results open the possibility that NLRP1 could function as a direct cytosolic sensor of other pathogen-derived proteases. More broadly, by adding direct proteolytic cleavage to the existing ligand-receptor models for NLR activation, our results also illustrate the remarkable adaptability of the NLR architecture to function as pathogen-detectors in host defense.

## Results

### Mouse NLRP1B is cleaved by LF

**Figure 1:**
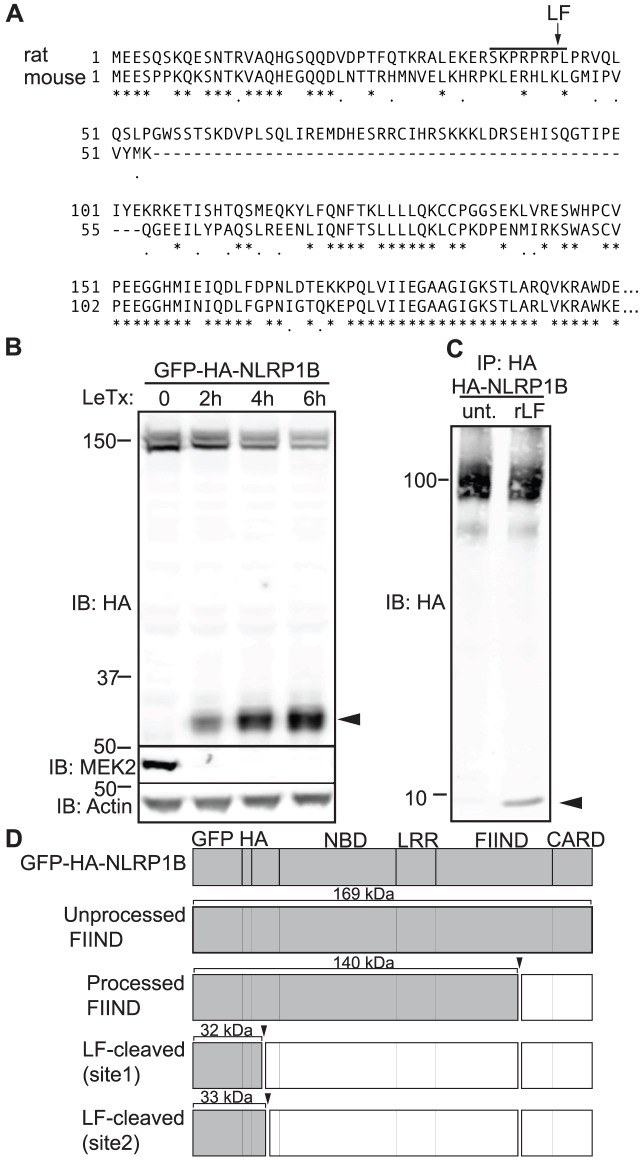
Murine NLRP1B from 129S1 mice is cleaved directly by LeTx. A) Protein sequence alignment of the N-terminal region of murine NLRP1B (129S1 allele) and rat NLRP1 (Fischer/CDF allele) was determined by ClutalW with a BLOSUM series matrix. The LF cleavage motif and cleavage site are identified in the rat allele by the bar and arrow above the rat sequence. B) GFP-HA-NLRP1B was transfected into HEK 293T cells and then treated with 1 µg/ml LeTx over the indicate time points followed by immunoblotting (IB) for HA on non-boiled lysates, and boiled lysates when probed with MEK2 and beta-actin antibodies. The arrow-head refers to the LeTx-dependent N-terminal cleavage fragment. C) HA-NLRP1B expressed in 293T cells, immunoprecipitated (IP) with anti-HA beads, and treated with recombinant LF (rLF) for 2 h, followed by immunoblotting for HA. D) Graphic representation of the GFP-HA-NLRP1B construct and annotated functional domains. The different forms of NLRP1B observed are shaded in gray along with their predicted molecular weights, when immunoblotted with an anti-HA antibody.

The N-termini of mouse NLRP1B and rat NLRP1 were recently reported to be cleaved by LF [41], [42]. Interestingly, these proteins do not exhibit much similarity in the region surrounding the cleavage site (Fig. 1A), whereas the rest of the protein is highly conserved between mice and rats (37% amino acid identity from position 1–54 vs. 70% identity from residue 55 to the C-terminus). The N-terminal fragment produced by LF is under 10 kDa and appears to be unstable, making it difficult to detect by conventional western blotting techniques in cell lysates. Thus, to confirm that mouse NLRP1B is cleaved by LF, we augmented the mass of the putative N-terminal fragment by 29 kDa by fusing full-length NLRP1B to enhanced green fluorescent protein (EGFP) and a hemagglutinin (HA) affinity-tag. We transfected this construct into HEK 293T cells and then treated the cells with LeTx. As reported previously, NLRP1B constitutively but only partially auto-processes its FIIND domain in untreated cells, resulting in a loss of 29 kDa from the C-terminus, and producing a doublet of 140 kDa and 169 kDa that we will refer to as the ‘processed’ and ‘unprocessed’ forms of NLRP1B, respectively (Fig. 1B and 1D) [24]. After LeTx addition, an N-terminal fragment smaller than 37 kDa, but larger than EGFP-HA alone (29 kDa), begins to accumulate inside cells. To distinguish LF-dependent cleavage from auto-processing of the FIIND domain, we will refer to the LF-dependent fragments as ‘cleaved’ (as opposed to ‘processed’) NLRP1B (Fig. 1D). With kinetics corresponding to the appearance of the cleaved N-terminal fragment, the amount of the detectable uncleaved NLRP1B decreased over time, consistent with removal of the N-terminal tag. The LF-dependent cleavage of NLRP1B is not complete even after 6 hours, and thus occurs much more slowly than the LF-dependent cleavage of the MAP kinase kinase MEK2, a canonical LF substrate, which appears complete within 2 hours (Fig. 1B).

To test if LF cleaves NLRP1B directly, without the GFP fusion, we expressed an HA-tagged NLRP1B in 293T cells, immunoprecipitated NLRP1B, and then treated the purified protein with recombinant LF *in vitro*. In the sample treated with LF, a fragment smaller than 10 kDa is produced (Fig. 1C), suggesting that LF can cleave mouse NLRP1B directly near the N-terminus, confirming recent findings [42].

### Cleavage is required for LF activation of NLRP1B

**Figure 2:**
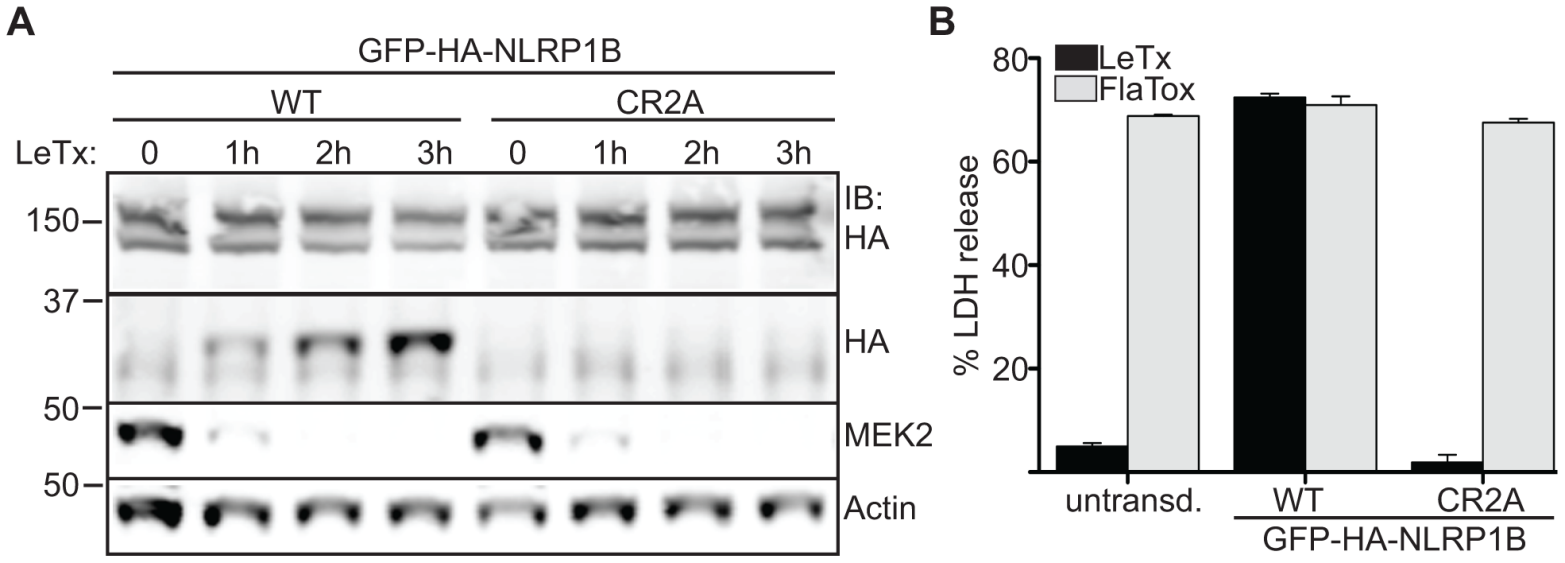
Mouse NLRP1B cleavage by LF is required for inflammasome activation. A) Both WT and CR2A GFP-HA-NLRP1B were transfected into 293T cells and then treated with LeTx for the indicated times, and cleavage was monitored by immunoblotting with indicated antibodies. B) Immortalized macrophages from a C57BL6 mouse were transduced with both forms of GFP-HA-NLRP1B and then treated with LeTx or LFn-Fla+PA (FlaTox). Pyroptosis was assayed by LDH release and normalized to complete detergent lysis. Error bars represent plus and minus one standard deviation from the mean.

Even though the cleavage site in rat NLRP1 is not well-conserved in mouse NLRP1B (Fig. 1A), two sequences can be found in the N-terminus of mouse NLRP1B that partially fit the previously established consensus specificity of LF [42] (Fig. S1A). For clarity, we refer to the putative site nearest to the N-terminus (cleavage after K38) as site-1, and the C-terminal site (cleavage after K44) as site-2 (Fig. 1D and S1A). These two sites were also identified as putative LF cleavage sites in a recent study [42], but their functional importance was not addressed. We attempted to generate cleavage resistant (CR) forms of NLRP1B by mutating each site. We made a variety of amino acid substitutions at site-1 (CR1A-D) and site-2 (CR2A-C) (Fig. S1A), utilizing residues that have previously been used to render MKK3 and MKK6 cleavage resistant [43], or residues not found in LF consensus sites [44], [45]. These mutants were transfected into 293T cells, and cells were then treated with LeTx and assayed for cleavage. Mutation of cleavage site-2 produced a cleavage-resistant form, despite the fact that site-1 is intact in this mutant (Fig. 2A S1A–C). By contrast, mutation of cleavage site-1 had little or no effect on NLRP1B cleavage (Fig. S1A–C). When *Casp1* and *Il1b* cDNA expression vectors were cotransfected into this same 293T system, only CR2A and CR2B were defective for induction of IL-1β processing into p17 above the basal processing induced by CASP1 and NLRP1B prior to stimulation (Fig. S1B–C). Thus, while confirming the previous finding that both site-1 and site-2 of mouse NLRP1B can be cleaved by LF [42], these results suggest that site-2 is the predominant LF target within NLRP1B in cells.

We tested the ability of the CR2A NLRP1B mutant to form an inflammasome capable of promoting pyroptosis. In these experiments, we used immortalized macrophages from a C57BL/6 (B6) mouse, because the endogenous B6 allele of NLRP1B is not responsive to LeTx. As expected, immortalized B6 macrophages transduced with a retroviral construct expressing the wild-type 129S1 allele of NLRP1B became sensitive to LeTx and underwent pyroptosis, as assessed by release of cytosolic lactate dehydrogenase (LDH) into the supernatant (Fig. 2B). By contrast, transduction of B6 macrophages with the CR2A NLRP1B mutant did not confer any measurable sensitivity to LeTx over the same time period. This difference in responsiveness is not due to differences in expression of the NLRP1B alleles (Fig. S2A). B6 cells harbor a functional NAIP5 inflammasome; thus, as a further control, the NLRP1B-transduced cells can be tested for inflammasome responses to the cytosolic presence of flagellin. We therefore delivered flagellin to the cytosol, via the protective antigen translocation channel used by lethal factor, as a fusion to the translocation signal in LF (dubbed ‘FlaTox’) [46] (Fig. 2B). Cells transduced with wild-type and CR2A NLRP1B were equally susceptible to FlaTox, indicating that they expressed functionally equivalent levels of anthrax toxin receptor, CASP1, and downstream effectors required for pyroptosis. These data demonstrate that the ability of mouse NLRP1B to respond to LF correlates with the ability of LF to cleave NLRP1B at its N-terminus.

### LF, expressed in the cytosol in the absence of PA, is sufficient to activate NLRP1B

The ability of LF to cleave and activate NLRP1B has only been tested in the presence of PA, since PA is typically required in order to deliver LF to the cytosol. It is therefore unclear if PA is only necessary for the translocation of LF in to the cytosol, or if it is also required for NLRP1B activation. We decided to test the ability of LF expression to induce pyroptosis and cytokine secretion in B6 and 129 immortalized macrophage-like cell lines with a Tet-On inducible vector. We transduced these cell lines with a lentiviral Tet-On GFP or LF expression vector and then treated the transduced cells with doxycycline to induce GFP or LF expression. LF expression was able to consistently induce pyroptosis in 129 (NLRP1B LeTx-responsive) cells but not B6 (NLRP1B LeTx-nonresponsive) cells (Fig. S5A). Further addition of PA had no additional effect on pyroptosis induction. Similar results were obtained when IL-1β production was used to monitor NLRP1B activation (Fig. S5B). These results show that the cytosolic presence of LF is sufficient to activate NLRP1B and that additional putative signals provided by PA pore formation are not required.

### Cleavage of NLRP1B is sufficient for inflammasome activation

**Figure 3:**
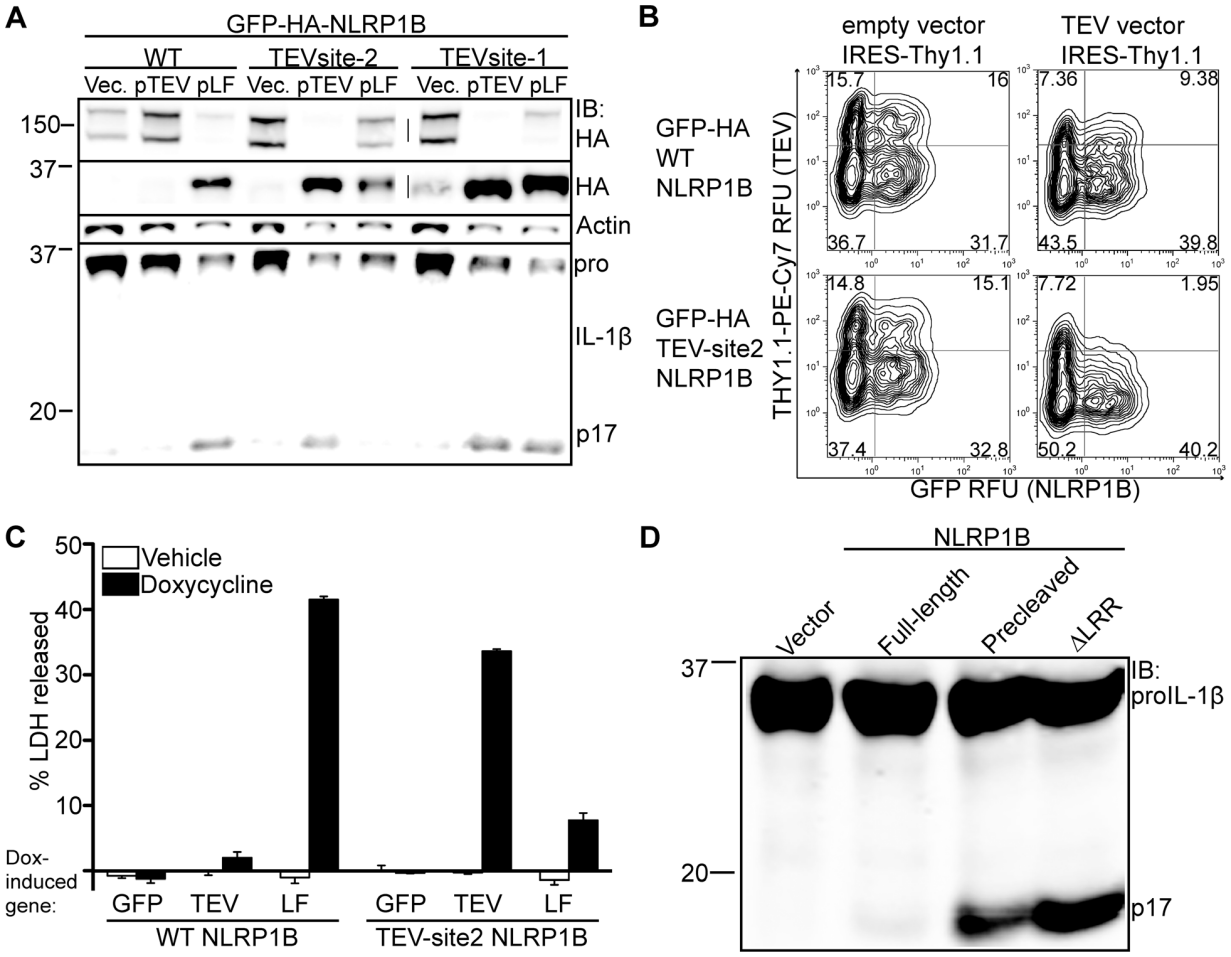
Cleavage of NLRP1B is sufficient to promote inflammasome activation. A) 293T cells were transfected with WT, TEV-site2 or TEV-site1 GFP-HA-NLRP1B along with empty vector, TEV expression vector, or a LF expression plasmids. In all conditions cells were also co-transfected with *Casp1* and *Il1b* expression vectors. Cleavage of GFP-HA-NLRP1B and IL-1β was determined 24 h post transfection. B) Immortalized B6 macrophages were transduced with a retrovirus encoding the indicated GFP-HA-NLRP1B form followed by a sequential transduction with a TEV-expression retrovirus co-expressing THY1.1. Percent transduction was determined by measuring expression of the respective retroviral integration markers (GFP and anti-THY1.1-PE-Cy7) by flow cytometry, and are expressed in relative fluorescent units (RFU). The numbers within each quadrant represent the percentage of live cells within the respective quadrant. C) RAW264.7 macrophages were transduced with GFP-HA-NLRP1B and a Tet-On construct expressing the indicated gene. Cells were treated with 5 µg/ml doxycycline for 20 h and supernatants were assayed for LDH release. D) 293T cells were transfected with empty vector, FL-NLRP1B-HA, the truncated NLRP1B-HA, or ΔLRR HA-NLRP1B, along with *Casp1* and *Il1b* and assayed by immunoblotting.

Together the above results suggest that mouse NLRP1B requires direct cleavage in order to be activated by LF, but it is difficult to rule out the formal possibility that the CR2A mutant is misfolded or is otherwise non-functional for reasons unrelated to its resistance to cleavage by LF. Moreover, the above experiments did not address whether cleavage alone is sufficient for activation of NLRP1B. For example, LF may have other substrates that must be cleaved in addition to NLRP1B, or LF itself could provide a ligand-like signal for the cleaved NLRP1B receptor. To address these possibilities, we replaced the predicted LF cleavage sites-1 and -2 in NLRP1B with a Tobacco Etch Virus (TEV) NIa protease cleavage-site (Fig. S1A). TEV protease was selected because it has no known endogenous substrates in mouse or human cells. We transfected 293T cells with plasmids expressing the wild-type and TEV-site forms of NLRP1B, along with plasmids encoding CASP1, pro-IL-1β, and either LF or TEV protease. As expected, wild-type NLRP1B was cleaved only in the presence of LF, and this coincided with the generation of mature IL-1β (Fig. 3A). Importantly, NLRP1B harboring a target sequence for TEV protease in place of the LF target sequence at site-2 (TEV-site2 NLRP1B) was cleaved efficiently by TEV protease, and this cleavage was sufficient to promote IL-1β processing. Consistent with the relatively low sequence specificity of LF, the TEV-site2 NLRP1B protein was also cleaved by LF, but this cleavage was inefficient as most of the NLRP1B remained uncleaved, and IL-1β was not efficiently processed. Cleavage of TEV-site1 also was sufficient to induce IL-1β processing, but this occurred upon expression of either LF or TEV proteases. Furthermore, the TEV-induced cleavage at site-1 produced a fragment of NLRP1B that was smaller than the fragment produced by LF expression (Fig. 3A and S1D). Consistent with the mutagenesis experiments shown in Fig. 2, this observation may indicate that LF prefers to cleave at site-2, which is still present in the TEV-site1 NLRP1B protein. Taken together, these results suggests that cleavage of NLRP1B at either site-1 or site-2 is sufficient to induce inflammasome activation independently of other LF-dependent cellular effects.

We also confirmed that cleavage of NLRP1B is sufficient to induce pyroptosis in macrophages cell lines. We transduced immortalized B6 macrophages with two different retroviral vectors, one expressing GFP-NLRP1B and the other expressing TEV protease with an IRES-Thy1.1 expression marker. If cleavage is sufficient to activate NLRP1B, it is expected that only cells expressing both components would undergo pyroptosis and therefore be underrepresented in the live population of cells. We analyzed the percentage of cells that contained both retroviruses by measuring THY1.1 surface-expression and GFP fluorescence by flow cytometry. As expected, an underrepresentation of the THY1.1 and GFP double-positive population was specifically seen in cells transduced with TEV and TEVsite2-NLRP1B, while the frequency of THY1.1+ cells was similar in cells that where negative for both forms of NLRP1B (Fig. 3B).

To further confirm that cleavage of NLRP1B is sufficient for inflammasome activation, we transduced RAW 264.7 cells with retroviral vectors encoding various NLRP1B alleles, as well as a lentiviral Tet-On vector that inducibly expresses GFP, TEV-protease or LF after exposure of cells to doxycycline. In this system, TEV expression induced high levels of LDH release only for cells expressing TEVsite2-NLRP1B. As expected, since RAW cells express an endogenous functional allele of NLRP1B, LF induced pyroptotic lysis of cells expressing either wild-type or TEVsite2-NLRP1B (Fig. 3C). The percent LDH release was generally consistent with the percentage of cells that expressed both constructs (Fig. S2B). These data demonstrate that cleavage of NLRP1B is sufficient to activate this inflammasome in macrophages and cause pyroptosis.

### No apparent role for the N-terminal NLRP1B cleavage fragment

We next tested whether the N-terminal fragment generated by cleavage of NLRP1B by LF at site-2 must be present along with the corresponding C-terminal fragment. We generated a construct to express a ‘pre-cleaved’ C-terminal fragment by deleting residues 1–44 of full-length NLRP1B and replacing amino acid 45 (leucine) with an initiator methionine. The resulting C-terminal fragment contains all known functional domains of NLRP1B (Fig. 1D). In the 293T cell system, high levels of spontaneous IL-1β cleavage was observed upon expression of the precleaved NLRP1B. The activity of pre-cleaved NLRP1B was comparable to that of a ΔLRR mutant, a form of NLRP1B that is known to be constitutively active (Fig. 3D and S3C) [47]. When the N-terminal fragment (amino acids 1–44) was coexpressed with the precleaved C-terminal fragment, no change in the amount of IL-1β processing was observed (Fig. S3B), suggesting it is neither necessary nor inhibitory when expressed in trans. For all of these experiments, the differences in the amount of IL-1β cleavage were not explained by differences in expression of NLRP1B (Fig. S3A–C).

### Proteasome inhibitors and FIIND processing do not affect LF-dependent cleavage

**Figure 4:**
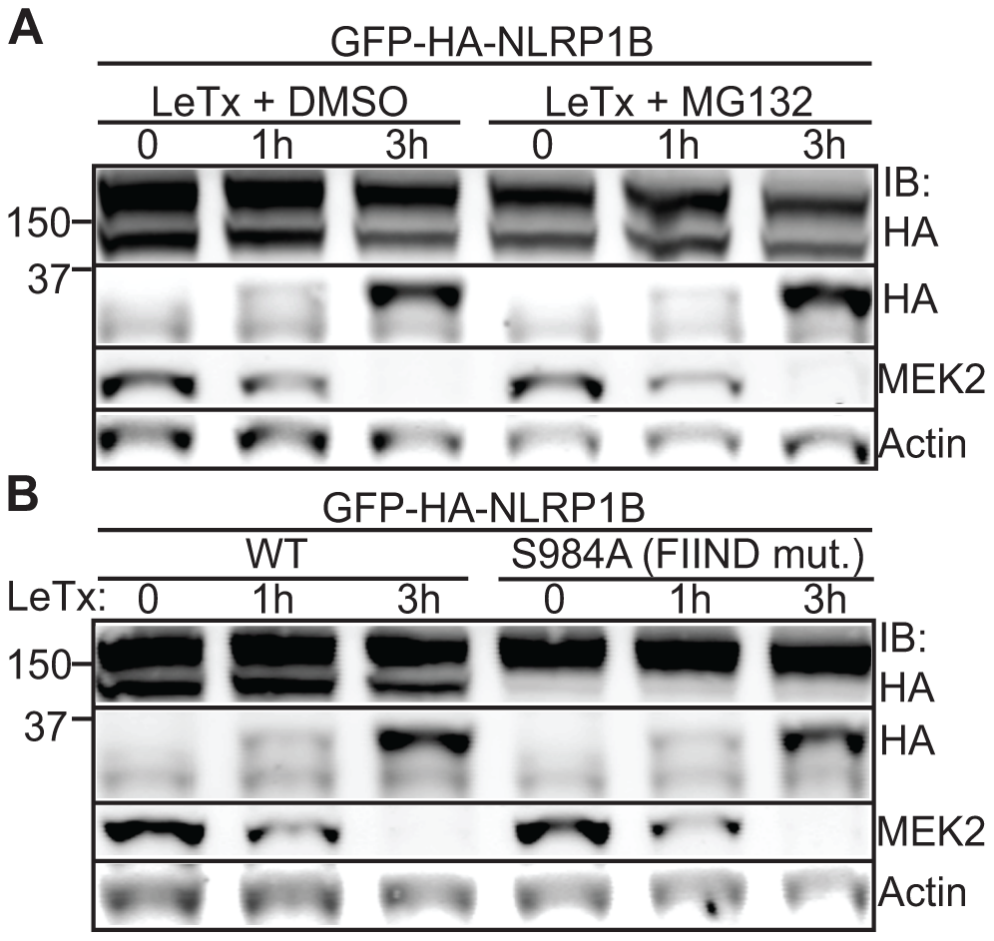
Proteasome inhibition and FIIND-processing do not affect NLRP1B cleavage by LF. A) 293T cells expressing GFP-HA-NLRP1B were co-treated with 1 µg/ml LeTx and 10 µM MG132 (proteasome inhibitor) or the DMSO vehicle and assayed for cleavage. B) Cleavage susceptibility of WT and S984A (FIIND mutant) GFP-HA-NLRP1B was determined in 293T cells at the indicated time points.

NLRP1B inflammasome activation can be blocked by proteasome inhibitors, an effect that is observed with multiple inhibitors and is specific to the NLRP1B inflammasome and not the NAIP/NLRC4 inflammasome [37], [39]. The mechanism by which proteasome inhibitors affect NLRP1B inflammasome activation is not currently known. Therefore, we tested whether the proteasome inhibitor MG132 blocked NLRP1B cleavage. In the 293T system, an equivalent amount of cleaved of NLRP1B occurred in the presence of MG132 and its vehicle (Fig. 4A), suggesting NLRP1B cleavage is not the step at which MG132 interferes with NLRP1B activation (Fig. S4A).

The FIIND of NLRP1B contains a ZU-5/UPA-like domain that can auto-process, and this auto-processing is required for NLRP1B activation [23], [24]. We tested if auto-processing at the FIIND region is prerequisite for N-terminal cleavage by LF. We tested the FIIND mutant S984A, which cannot auto-process, and found it to be indistinguishably sensitive to LeTx cleavage as wild-type NLRP1B (Fig. 4B). Thus FIIND auto-processing appears to be required for a downstream step in NLRP1B activation (Fig. S4B), and is not required for NLRP1B to be sensitive to LeTx cleavage.

## Discussion

Activation of the NLRP1B inflammasome by LeTx is an important resistance mechanism during *Bacillus anthracis* infections in mice [17], [18]. However the question of how NLRP1B senses the protease activity of LF remains unresolved. Here we investigated the molecular mechanism by which the protease activity of *B. anthracis* lethal toxin could be detected by NLRP1B. Our studies provide a clear molecular mechanism for how a pathogen-encoded activity (or ‘pattern of pathogenesis’ [3]) can be sensed by the innate immune system.

Two recent studies by Moayeri and colleagues provided a considerable advance in our understanding of NLRP1B activation by LeTx [41], [42]. These two studies showed that both rat and mouse NLRP1 can be cleaved near the N-terminus by LF, and that mutation of the cleavage site abolished responsiveness of rat NLRP1 to LF. While these findings strongly suggest that direct cleavage of rat NLRP1 could be its mechanism of activation, the functional role of cleavage of mouse NLRP1B was not addressed, and it is also possible that mutation of the cleavage site blocked activation of rat NLRP1 by affecting the folding or assembly of NLRP1. Most significantly, the question of whether cleavage of NLRP1B was sufficient for its activation has not been addressed. This question is especially important to address because LT has been shown to have complex effects on cells, including disruption of MAP kinase signaling [43], [48], [49], that could conceivably play a role in NLRP1B activation. Moreover, several other cellular functions, such as proteasome activity and N-end rule degradation pathways, have been implicated in NLRP1B activation [37], [39], [40]. Therefore, to demonstrate that cleavage of NLRP1B is sufficient to induce inflammasome activation, we engineered an allele of NLRP1B that could be activated by the heterologous TEV protease. This protease is not known to have endogenous substrates in mouse or human cells, so is likely to exert its effects solely via direct cleavage of the engineered NLRP1B protein. Indeed, the TEV protease did not activate NLRP1B unless NLRP1B contained a site that could be cleaved by TEV (Fig. 3A). Recent data have suggested that mouse NLRP1B can be cleaved at two distinct sites by LF [42], but our cleavage site mutants and TEV-site forms of the receptor are most consistent with site-2 (cleavage between residues 44–45) being the predominant cleavage site. Interestingly, this site coincides with the same amino acid position as the LF cleavage site in rat NLRP1, even though the sequences of the two sites are not conserved (Fig. 1A). The low degree of target sequence specificity exhibited by LF may have allowed the sequence of the cleavage site in NLRP1 to diverge without losing responsiveness to LF. The position within NLRP1 at which LF cleaves may be determined in part by interactions between LF and regions of NLRP1 outside of the cleavage site. Indeed, similar non-cleavage-site interactions appear to control the specificity of LF for its other known substrates, the MAPKKs [9], [10]. The divergence of the amino acid sequence of the N-terminus of NLRP1B is interesting given the high degree of conservation in the rest of the protein. This divergence may be due to random drift of a structurally unconstrained domain, or alternatively, the divergence may reflect evolutionary pressure for NLRP1 to be recognized by other pathogen-encoded proteases. Notably, our data suggest that cleavage outside of the primary LF target site (e.g., at site-1) can also activate NLRP1B (Fig. 3A), although it is unclear if LF can cleave and activate NLRP1B at this position. In addition, our data suggest that cleavage of NLRP1B does not necessarily have to be complete to be sufficient to permit inflammasome assembly and CASP1 activation. Taken together, these observations suggest that NLRP1B could be responsive to proteases from other pathogens even if these proteases cleave NLRP1B at different sites with low efficiency. Indeed, countless pathogens, including bacteria, viruses and parasites, depend on cytosolically-localized proteases for virulence [50]–[53]. Therefore the presence of cytosolic proteases could be considered a ‘pattern of pathogenesis’ [3], that could be detected by NLRP1 proteins to allow the innate immune system to discriminate pathogenic and harmless microbes. The divergence of rat and mouse NLRP1 may thus reflect evolution under the selective pressure imposed by distinct sets of pathogens in the two different rodents species. It will be interesting to determine if other proteases can activate rodent NLRP1s.

The detection of protease activity by NLRP1B represents a fundamentally distinct mode of pathogen recognition in vertebrates as compared to the classic mode of direct recognition of PAMPs observed with most innate immune receptors of the TLR, NLR and RLR families. The N-terminus of NLRP1B appears to function to detect LF activity in a manner analogous to the ‘decoy’ model [54], which has been previously proposed to explain detection of certain pathogen effectors by plant NLRs. The proteolytic mechanism by which NLRP1B is activated represents one of the few examples in mammals in which a molecular mechanism has been established for how an innate immune sensor can respond to a pathogen-encoded activity.

It is currently unknown how cleavage of the N-terminus results in structural changes that lead to NLRP1B activation. A simple model is that the N-terminus of NLRP1B mediates an auto-inhibitory intramolecular interaction, perhaps via an interaction with the LRR domain, which is known also to be required for auto-inhibition of NLRP1B (Fig. 3D) [47]. An alternative model that is not excluded by our data is that the removal of the original N-terminus allows the neo-N-terminus to provide a positive signal to activate NLRP1B. More complicated models involving interactions with other proteins can also be envisaged. We did not observe an inhibitory role of the N-terminal fragment when expressed in trans (Fig. S3). This suggests that the N-terminus is necessary to maintain NLRP1B in a conformation that is inactive, but can only do so when the N-terminus is covalently attached to the rest of NLRP1B.

In addition to proteolytic cleavage by LF, additional layers of NLRP1B regulation appear to exist. For example, FIIND auto-processing is required for NLRP1B activity, for reasons that remain poorly understood [24]. Since we found that FIIND auto-processing mutants are still cleaved by LF, the role of FIIND auto-processing appears not to be to render NLRP1B susceptible to cleavage by LF. Further complexities in NLRP1B activation are also suggested by the observation that proteasome and N-end rule pathway inhibitors appear to specifically prevent NLRP1B-dependent CASP1 activation [37], [39], [40]. Even though previous studies have shown that proteasome inhibitors do not block cleavage of MAPKK by LF [37], [39], we tested if proteasome inhibition might affect cleavage of NLRP1B, which appears to be a less optimal substrate than the MAPKKs. However, we observed no effect of the proteasome inhibitor MG132 on the ability of LF to cleave NLRP1B. Thus it remains unclear how this inhibitor specifically blocks the NLRP1B inflammasome and not other inflammasomes. Models that attempt to explain the mechanism of NLRP1B are further complicated by other unique features of NLRP1B. For example, ATP binding to the NBD of NLRP1B is not necessary for inflammasome activation, and mutants of NLRP1B that are unable to bind ATP are actually constitutively active [55]. This is contrary to what is known for other mammalian NLRs, where ATP binding appears to be required for oligomerization and downstream signaling [21]. Furthermore, gross truncations of NLRP1B can also lead to constitutively active forms of NLRP1B that contain only the very C-terminal CARD and a portion of the FIIND [47]. Thus, other disturbances, by proteolysis or by other means, to the overall structure of NLRP1B could lead to loss of the conformation that mediates auto-inhibition.

In general, the molecular conformational changes that occur in NLRs as they transition from an inactive to an active state are poorly understood. Thus, our studies of NLRP1B provide an important point of comparison that helps us to develop a broader understanding of the NLR class of innate immune sensors and the mechanisms of their activation.

## Materials and Methods

### Ethics statement

This study was carried out in strict accordance with the recommendations in the Guide for the Care and Use of Laboratory Animals of the National Institutes of Health. The protocol was approved by the Animal Care and Use Committee at the University of California, Berkeley (MAUP #: R301-0313BRC).

### Plasmids and constructs

HA-NLRP1B was amplified from a *Nlrp1b* (DQ117584.1) cDNA (gift of E. Boyden and B. Dietrich, Harvard Medical School) with primers 1–2 and cloned into pCMSCV-IRES-hCD4 using the XhoI and NotI restriction sites (Fig. S5). A construct for expression a GFP-HA-NLRP1B fusion was created by amplifying HA-NLRP1B with primers 3–4 and cloning the resulting PCR product into MSCV downstream of and in-frame with GFP using NotI and SalI sites. TEV expression constructs were created by amplifying a His6-TEV ORF with the primers 7–9 and 8–9 into pCMSCV-IRES-Thy1.1 and pFG12-rtTA-IRES-Thy1.1, respectively. LF was similarly cloned into the same vectors as TEV, but with the primers 10–11 and 12–13 using a template provided by Bryan Krantz (UC Berkeley). Mutagenesis of *Nlrp1b* was performed using Quickchange (Stratagene/Agilent), but modified by substituting the *Pfu* polymerase for PrimeSTAR HS (TAKARA/Clonetech). The primers used are listed in Fig. S6.

### Cell culture

HEK293T (ATCC) cells were grown in complete media (DMEM, 10%FBS, 100 U/ml Penicillin, 100 µg/ml Streptomycin, and supplemented with 2 mM L-glutamine). RAW 264.7 and immortalized B6 macrophages were grown in complete media (RPMI 1640, 10%FBS, 100 U/ml Penicillin, 100 µg/ml Streptomycin).

### DNA transient transfections

HEK 293T cells were seeded the day prior to transfection at a density of 1.5×10^5^ cells/well in a 24-well plate with complete media. DNA complexes were made with Lipofectamine 2000 (Invitrogen) according to manufactures instructions and overlaid on cells for 24–36 hours.

### Western blots

Cells were lysed in RIPA buffer supplemented with 1 mM PMSF and 1×X Complete Protease Inhibitor Cocktail (Roche). Lysates were spun down at max speed at 4C for 20 min and supernatants were mixed with 6× Laemmli sample buffer. To detect full length NLRP1B, lysates were incubated at room temperature prior to SDS-PAGE. To analyze all other proteins, including the N-terminally cleaved form of NLRP1B, samples were boiled for 10 min prior to separation. SDS-PAGE was performed with Novex BisTris gel system according to manufacturer instructions (Invitrogen). Separated proteins were transferred on to Immobilon-FL PVDF membranes. Membranes were blocked with Odyssey blocking buffer (Licor). The following antibodies were used for the following antigens: HA mAB 3F10 (Roche), MEK-2 SC-13115 (Santa Cruz), Beta Actin SC-4778 (Santa Cruz), IL-1B AF-401-NA (R&D systems). Secondary antibodies anti-rat, mouse and goat were all conjugated to Alexa Flour-680 (Invitrogen).

### Immunoprecipitation and LF *in vitro* cleavage assay

Transfected cells were lysed in a non-denaturing buffer (1% NP-40, 137 mM NaCl, 2 mM EDTA, 20 mM Tris pH 8 supplemented with protease inhibitors). Cleared lysates were bound to EZview Red Anti-HA Affinity Gel (Sigma) washed four times with lysis buffer, once in LF cleavage buffer (10 mM NaCl, 5 uM ZnSO4, 10 mM HEPES pH 7.4), and resuspended back into cleavage buffer. One microgram of recombinant LF was added to immunoprecipitated NLRP1B and incubated at 37°C for 2 hours, and analyzed by western blotting as described above.

### Cytotoxicity/Pyroptosis assay and IL-1β secretion

Macrophages were seeded one day prior to treatment in a 96well plate at 5×10^4^ cell/well in RPMI media without phenol red. The next day cells were treated with LeTx 1 µg/ml, FlaTox 1 µg/ml [46], or doxycycline at 5 µg/ml in ethanol for the indicated time, and spun down at 400×*g*. For IL-1β release cells were pretreated/cotreated with 1 µg/ml of Pam3CSK4. Supernatants were removed and assayed for LDH and IL-1β release as described previously [56].

## Supporting Information

Figure S1 **Predicted LF cleavage-site mutation comparison.** A) LF cleavage-site1 predicts cleavage between K38-39 and the motif surrounding this site was progressively mutated away from the consensus motif. Cleavage-site 2 predicts cleavage between K44 and L45, and this site was muted only at residues immediately surrounding the site. TEV cleavage sites were introduced to produce cleavage after residue 38 for site-1, and residue 44 for site-2. B) Cleavage site-1 mutant series (CR1A-D)) and CR2A were transfected into 293T cells and treated with 1 µg/ml LeTx for 4 h, analyzed by western blotting with the indicated antibodies. The bottom blot was done in the presence of *Casp1* to determine the extent of IL-1β processing. C) WT, CR1D, CR2A-C GFP-HA-NLRP1B were transfected into 293T cells and treated with 1 µg/ml LeTx for the indicated time points and cleavage and IL-1β processing was assayed by western blotting. D) WT and TEV-site1 GFP-HANLRP1B were cotransfected with TEV or empty expression vector into 293T cells. Thirty-six hours post transfection cells were treated with LeTx for 4 h, then lysed and analyzed by immunoblotting with an anti-HA antibody.
(TIF)

Figure S2 **Transduction efficiency is the same in macrophage cell lines.** A) Immortalized B6 macrophages were transduced with WT and CR2A GFP-HA-NLRP1B. Expression and cleavage of each NLRP1B was determined by western blotting. Glycine (5 mM) was added 1 hour post the addition of LeTx to block lysis of cells in the 2 h and 3 h time points. B) Percent transduction of RAW 264.7 macrophages was determined by measuring THY1.1 surface expression for the Tet-On vector, and GFP expression for the NLRP1B vector under non-inducing conditions by flow cytometry. GFP and anti-THY1.1-PE-Cy7 fluorescence are expressed in relative fluorescence units (RFU). The numbers within each quadrant represent the percentage of live cells within the respective quadrant.
(EPS)

Figure S3 **NLRP1B's N-terminal fragment has no role in inflammasome activation when expressed in trans.** A) Expression of FL, precleaved and ΔLRR mutants of NLRP1B were determined by anti-HA immunoblotting. B) 293T cells were cotransfected with C-terminally HA tagged WT or precleaved NLRP1B along with *Casp1* and *Il1b* expression constructs. The N-terminal fragment (residues 1–44) fused to GFP-HA was co-transfected with the C-terminal fragments and assayed for IL-1β 24 h post-transfection. C) Processing of IL-1β in 293T cells was determined to be dependent on NLRP1B expression and the catalytic activity of CASP1.
(TIF)

Figure S4 **MG132 blocks NLRP1B activity and FIIND processing is required in 293T cells.** A) IL-1β processing was analyzed in 293T cells expressing GFP-HA-NLRP1B, *Casp1*, *Il1b* and treated with MG132 (proteasome inhibitor) and LeTx for the indicated time points. B) The necessity of FIIND domain processing for NLRP1B activation in 293T cells was determined by measuring IL-1β processing in cells expressing GFP-HA-NLRP1B, *Casp1*, *Il1b*, after treatment with LeTx for the indicated time points.
(TIF)

Figure S5 **LF expression is sufficient to induce pyroptosis and IL-1β in 129 macrophages.** A,B) Immortalized C57BL/6 (B6) and 129 macrophages were transduced with a Tet-On construct expressing the GFP or LF. Cells were treated with 1 µg/ml Pam3CSK4, 5 µg/ml doxycycline and 1 µg/ml PA for 20 h and supernatants were assayed for LDH release (A) and IL-1β release (B) into the supernatant. Cells were also treated with 1 µg/ml LeTx and 5 µg/ml FlaTox for 4 h prior supernatant collection. Error bars represent plus and minus one standard deviation from the mean. ND stands for not determined.
(EPS)

Figure S6 **PCR primers used for cloning and mutagenesis.**
(EPS)

## References

[1] Takeuchi O, Akira S (2010) Pattern recognition receptors and inflammation. Cell 140: 805–820. 20303872 10.1016/j.cell.2010.01.022

[2] Janeway CA Jr (1989) Approaching the asymptote? Evolution and revolution in immunology. Cold Spring Harb Symp Quant Biol 54 Pt 1: 1–13. 10.1101/sqb.1989.054.01.003 2700931

[3] Vance RE, Isberg RR, Portnoy DA (2009) Patterns of pathogenesis: discrimination of pathogenic and nonpathogenic microbes by the innate immune system. Cell Host Microbe 6: 10–21. 19616762 10.1016/j.chom.2009.06.007 PMC2777727

[4] Fontana MF, Banga S, Barry KC, Shen X, Tan Y, et al (2011) Secreted bacterial effectors that inhibit host protein synthesis are critical for induction of the innate immune response to virulent Legionella pneumophila. PLoS Pathog 7: e1001289. 21390206 10.1371/journal.ppat.1001289 PMC3040669

[5] Shin S, Case CL, Archer KA, Nogueira CV, Kobayashi KS, et al (2008) Type IV secretion-dependent activation of host MAP kinases induces an increased proinflammatory cytokine response to Legionella pneumophila. PLoS Pathog 4: e1000220. 19043549 10.1371/journal.ppat.1000220 PMC2582680

[6] Boyer L, Magoc L, Dejardin S, Cappillino M, Paquette N, et al (2011) Pathogen-derived effectors trigger protective immunity via activation of the Rac2 enzyme and the IMD or Rip kinase signaling pathway. Immunity 35: 536–549. 22018470 10.1016/j.immuni.2011.08.015 PMC3258503

[7] Keestra AM, Winter MG, Auburger JJ, Frassle SP, Xavier MN, et al (2013) Manipulation of small Rho GTPases is a pathogen-induced process detected by NOD1. Nature 496: 233–237. 23542589 10.1038/nature12025 PMC3625479

[8] Duesbery NS, Webb CP, Leppla SH, Gordon VM, Klimpel KR, et al (1998) Proteolytic inactivation of MAP-kinase-kinase by anthrax lethal factor. Science 280: 734–737. 9563949 10.1126/science.280.5364.734

[9] Vitale G, Pellizzari R, Recchi C, Napolitani G, Mock M, et al (1998) Anthrax lethal factor cleaves the N-terminus of MAPKKs and induces tyrosine/threonine phosphorylation of MAPKs in cultured macrophages. Biochem Biophys Res Commun 248: 706–711. 9703991 10.1006/bbrc.1998.9040

[10] Chopra AP, Boone SA, Liang X, Duesbery NS (2003) Anthrax lethal factor proteolysis and inactivation of MAPK kinase. J Biol Chem 278: 9402–9406. 12522135 10.1074/jbc.M211262200

[11] Bardwell AJ, Abdollahi M, Bardwell L (2004) Anthrax lethal factor-cleavage products of MAPK (mitogen-activated protein kinase) kinases exhibit reduced binding to their cognate MAPKs. Biochem J 378: 569–577. 14616089 10.1042/BJ20031382 PMC1223970

[12] Bergsbaken T, Fink SL, Cookson BT (2009) Pyroptosis: host cell death and inflammation. Nat Rev Microbiol 7: 99–109. 19148178 10.1038/nrmicro2070 PMC2910423

[13] Boyden ED, Dietrich WF (2006) Nalp1b controls mouse macrophage susceptibility to anthrax lethal toxin. Nat Genet 38: 240–244. 16429160 10.1038/ng1724

[14] Friedlander AM (1986) Macrophages are sensitive to anthrax lethal toxin through an acid-dependent process. J Biol Chem 261: 7123–7126. 3711080

[15] Friedlander AM, Bhatnagar R, Leppla SH, Johnson L, Singh Y (1993) Characterization of macrophage sensitivity and resistance to anthrax lethal toxin. Infect Immun 61: 245–252. 8380282 10.1128/iai.61.1.245-252.1993 PMC302711

[16] Newman ZL, Printz MP, Liu S, Crown D, Breen L, et al (2010) Susceptibility to anthrax lethal toxin-induced rat death is controlled by a single chromosome 10 locus that includes rNlrp1. PLoS Pathog 6: e1000906. 20502689 10.1371/journal.ppat.1000906 PMC2873920

[17] Terra JK, Cote CK, France B, Jenkins AL, Bozue JA, et al (2010) Cutting edge: resistance to Bacillus anthracis infection mediated by a lethal toxin sensitive allele of Nalp1b/Nlrp1b. J Immunol 184: 17–20. 19949100 10.4049/jimmunol.0903114 PMC2811128

[18] Moayeri M, Crown D, Newman ZL, Okugawa S, Eckhaus M, et al (2010) Inflammasome sensor Nlrp1b-dependent resistance to anthrax is mediated by caspase-1, IL-1 signaling and neutrophil recruitment. PLoS Pathog 6: e1001222. 21170303 10.1371/journal.ppat.1001222 PMC3000361

[19] Tschopp J, Martinon F, Burns K (2003) NALPs: a novel protein family involved in inflammation. Nat Rev Mol Cell Biol 4: 95–104. 12563287 10.1038/nrm1019

[20] Ting JP, Lovering RC, Alnemri ES, Bertin J, Boss JM, et al (2008) The NLR gene family: a standard nomenclature. Immunity 28: 285–287. 18341998 10.1016/j.immuni.2008.02.005 PMC2630772

[21] von Moltke J, Ayres JS, Kofoed EM, Chavarria-Smith J, Vance RE (2012) Recognition of Bacteria by Inflammasomes. Annu Rev Immunol 31: 73–106. 23215645 10.1146/annurev-immunol-032712-095944

[22] Martinon F, Burns K, Tschopp J (2002) The inflammasome: a molecular platform triggering activation of inflammatory caspases and processing of proIL-beta. Mol Cell 10: 417–426. 12191486 10.1016/s1097-2765(02)00599-3

[23] D'Osualdo A, Weichenberger CX, Wagner RN, Godzik A, Wooley J, et al (2011) CARD8 and NLRP1 undergo autoproteolytic processing through a ZU5-like domain. PLoS One 6: e27396. 22087307 10.1371/journal.pone.0027396 PMC3210808

[24] Frew BC, Joag VR, Mogridge J (2012) Proteolytic processing of Nlrp1b is required for inflammasome activity. PLoS Pathog 8: e1002659. 22536155 10.1371/journal.ppat.1002659 PMC3334886

[25] Finger JN, Lich JD, Dare LC, Cook MN, Brown KK, et al (2012) Autolytic proteolysis within the function to find domain (FIIND) is required for NLRP1 inflammasome activity. J Biol Chem 287: 25030–25037. 22665479 10.1074/jbc.M112.378323 PMC3408201

[26] Zhao Y, Yang J, Shi J, Gong YN, Lu Q, et al (2011) The NLRC4 inflammasome receptors for bacterial flagellin and type III secretion apparatus. Nature 477: 596–600. 21918512 10.1038/nature10510

[27] Kofoed EM, Vance RE (2011) Innate immune recognition of bacterial ligands by NAIPs determines inflammasome specificity. Nature 477: 592–595. 21874021 10.1038/nature10394 PMC3184209

[28] Roberts TL, Idris A, Dunn JA, Kelly GM, Burnton CM, et al (2009) HIN-200 proteins regulate caspase activation in response to foreign cytoplasmic DNA. Science 323: 1057–1060. 19131592 10.1126/science.1169841

[29] Burckstummer T, Baumann C, Bluml S, Dixit E, Durnberger G, et al (2009) An orthogonal proteomic-genomic screen identifies AIM2 as a cytoplasmic DNA sensor for the inflammasome. Nat Immunol 10: 266–272. 19158679 10.1038/ni.1702

[30] Hornung V, Ablasser A, Charrel-Dennis M, Bauernfeind F, Horvath G, et al (2009) AIM2 recognizes cytosolic dsDNA and forms a caspase-1-activating inflammasome with ASC. Nature 458: 514–518. 19158675 10.1038/nature07725 PMC2726264

[31] Fernandes-Alnemri T, Yu JW, Datta P, Wu J, Alnemri ES (2009) AIM2 activates the inflammasome and cell death in response to cytoplasmic DNA. Nature 458: 509–513. 19158676 10.1038/nature07710 PMC2862225

[32] Ichinohe T, Pang IK, Iwasaki A (2010) Influenza virus activates inflammasomes via its intracellular M2 ion channel. Nat Immunol 11: 404–410. 20383149 10.1038/ni.1861 PMC2857582

[33] Jones JD, Dangl JL (2006) The plant immune system. Nature 444: 323–329. 17108957 10.1038/nature05286

[34] Chisholm ST, Coaker G, Day B, Staskawicz BJ (2006) Host-microbe interactions: shaping the evolution of the plant immune response. Cell 124: 803–814. 16497589 10.1016/j.cell.2006.02.008

[35] Axtell MJ, Staskawicz BJ (2003) Initiation of RPS2-specified disease resistance in Arabidopsis is coupled to the AvrRpt2-directed elimination of RIN4. Cell 112: 369–377. 12581526 10.1016/s0092-8674(03)00036-9

[36] Mackey D, Belkhadir Y, Alonso JM, Ecker JR, Dangl JL (2003) Arabidopsis RIN4 is a target of the type III virulence effector AvrRpt2 and modulates RPS2-mediated resistance. Cell 112: 379–389. 12581527 10.1016/s0092-8674(03)00040-0

[37] Fink SL, Bergsbaken T, Cookson BT (2008) Anthrax lethal toxin and Salmonella elicit the common cell death pathway of caspase-1-dependent pyroptosis via distinct mechanisms. Proc Natl Acad Sci U S A 105: 4312–4317. 18337499 10.1073/pnas.0707370105 PMC2393760

[38] Klimpel KR, Arora N, Leppla SH (1994) Anthrax toxin lethal factor contains a zinc metalloprotease consensus sequence which is required for lethal toxin activity. Mol Microbiol 13: 1093–1100. 7854123 10.1111/j.1365-2958.1994.tb00500.x

[39] Wickliffe KE, Leppla SH, Moayeri M (2008) Anthrax lethal toxin-induced inflammasome formation and caspase-1 activation are late events dependent on ion fluxes and the proteasome. Cell Microbiol 10: 332–343. 17850338 10.1111/j.1462-5822.2007.01044.x PMC2515708

[40] Wickliffe KE, Leppla SH, Moayeri M (2008) Killing of macrophages by anthrax lethal toxin: involvement of the N-end rule pathway. Cell Microbiol 10: 1352–1362. 18266992 10.1111/j.1462-5822.2008.01131.x PMC2500182

[41] Levinsohn JL, Newman ZL, Hellmich KA, Fattah R, Getz MA, et al (2012) Anthrax lethal factor cleavage of Nlrp1 is required for activation of the inflammasome. PLoS Pathog 8: e1002638. 22479187 10.1371/journal.ppat.1002638 PMC3315489

[42] Hellmich KA, Levinsohn JL, Fattah R, Newman ZL, Maier N, et al (2012) Anthrax lethal factor cleaves mouse nlrp1b in both toxin-sensitive and toxin-resistant macrophages. PLoS One 7: e49741. 23152930 10.1371/journal.pone.0049741 PMC3495862

[43] Park JM, Greten FR, Li ZW, Karin M (2002) Macrophage apoptosis by anthrax lethal factor through p38 MAP kinase inhibition. Science 297: 2048–2051. 12202685 10.1126/science.1073163

[44] Zakharova MY, Kuznetsov NA, Dubiley SA, Kozyr AV, Fedorova OS, et al (2009) Substrate recognition of anthrax lethal factor examined by combinatorial and pre-steady-state kinetic approaches. J Biol Chem 284: 17902–17913. 19359249 10.1074/jbc.M807510200 PMC2709388

[45] Turk BE, Wong TY, Schwarzenbacher R, Jarrell ET, Leppla SH, et al (2004) The structural basis for substrate and inhibitor selectivity of the anthrax lethal factor. Nat Struct Mol Biol 11: 60–66. 14718924 10.1038/nsmb708

[46] von Moltke J, Trinidad NJ, Moayeri M, Kintzer AF, Wang SB, et al (2012) Rapid induction of inflammatory lipid mediators by the inflammasome in vivo. Nature 490: 107–111. 22902502 10.1038/nature11351 PMC3465483

[47] Liao KC, Mogridge J (2009) Expression of Nlrp1b inflammasome components in human fibroblasts confers susceptibility to anthrax lethal toxin. Infect Immun 77: 4455–4462. 19651869 10.1128/IAI.00276-09 PMC2747971

[48] Ali SR, Timmer AM, Bilgrami S, Park EJ, Eckmann L, et al (2011) Anthrax toxin induces macrophage death by p38 MAPK inhibition but leads to inflammasome activation via ATP leakage. Immunity 35: 34–44. 21683629 10.1016/j.immuni.2011.04.015 PMC3889666

[49] Turk BE (2007) Manipulation of host signalling pathways by anthrax toxins. Biochem J 402: 405–417. 17313374 10.1042/BJ20061891

[50] Potempa J, Pike RN (2009) Corruption of innate immunity by bacterial proteases. J Innate Immun 1: 70–87. 19756242 10.1159/000181144 PMC2743019

[51] Toh EC, Huq NL, Dashper SG, Reynolds EC (2010) Cysteine protease inhibitors: from evolutionary relationships to modern chemotherapeutic design for the treatment of infectious diseases. Curr Protein Pept Sci 11: 725–743. 21235508 10.2174/138920310794557646

[52] Anderson J, Schiffer C, Lee SK, Swanstrom R (2009) Viral protease inhibitors. Handb Exp Pharmacol 2009: 85–110. 10.1007/978-3-540-79086-0_4 PMC7120715 19048198

[53] Li H, Child MA, Bogyo M (2012) Proteases as regulators of pathogenesis: examples from the Apicomplexa. Biochim Biophys Acta 1824: 177–185. 21683169 10.1016/j.bbapap.2011.06.002 PMC3232290

[54] van der Hoorn RA, Kamoun S (2008) From Guard to Decoy: a new model for perception of plant pathogen effectors. Plant Cell 20: 2009–2017. 18723576 10.1105/tpc.108.060194 PMC2553620

[55] Liao KC, Mogridge J (2012) Activation of the Nlrp1b Inflammasome by Reduction of Cytosolic ATP. Infect Immun 81: 570–9. 23230290 10.1128/IAI.01003-12 PMC3553809

[56] Lightfield KL, Persson J, Brubaker SW, Witte CE, von Moltke J, et al (2008) Critical function for Naip5 in inflammasome activation by a conserved carboxy-terminal domain of flagellin. Nat Immunol 9: 1171–1178. 18724372 10.1038/ni.1646 PMC2614210

