# Supplementary figures and images for: Direct Proteolytic Cleavage of NLRP1B Is Necessary and Sufficient for Inflammasome Activation by Anthrax Lethal Factor
Source: PLoS Pathog. 2013 Jun 20;9(6):e1003452. doi: 10.1371/journal.ppat.1003452 (PMC3688554; doi:10.1371/journal.ppat.1003452)

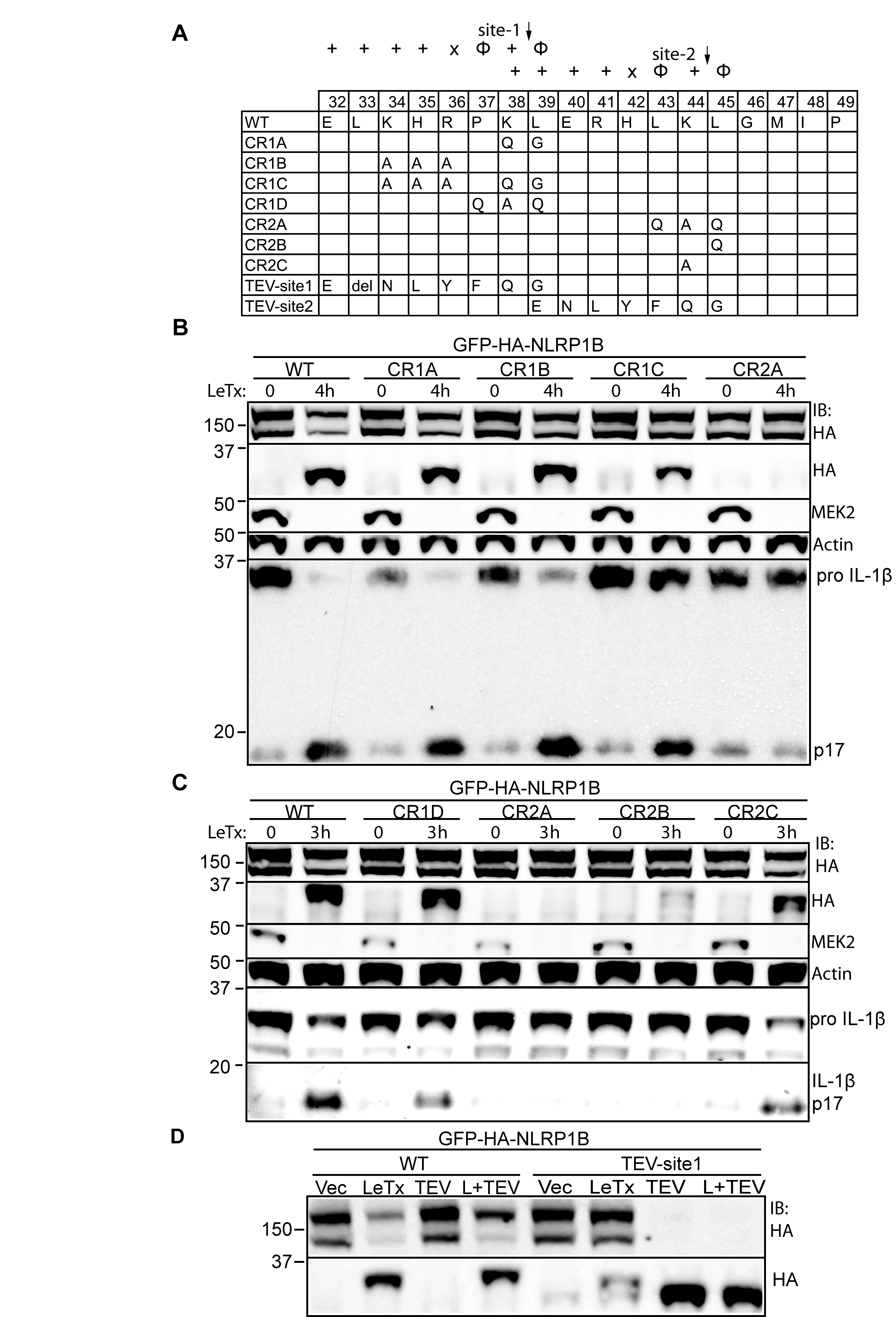

Supplement: Figure S1 — Predicted LF cleavage-site mutation comparison. A) LF cleavage-site1 predicts cleavage between K38-39 and the motif surrounding this site was progressively mutated away from the consensus motif. Cleavage-site 2 predicts cleavage between K44 and L45, and this site was muted only at residues immediately surrounding the site. TEV cleavage sites were introduced to produce cleavage after residue 38 for site-1, and residue 44 for site-2. B) Cleavage site-1 mutant series (CR1A-D)) and CR2A were transfected into 293T cells and treated with 1 µg/ml LeTx for 4 h, analyzed by western blotting with the indicated antibodies. The bottom blot was done in the presence of Casp1 to determine the extent of IL-1β processing. C) WT, CR1D, CR2A-C GFP-HA-NLRP1B were transfected into 293T cells and treated with 1 µg/ml LeTx for the indicated time points and cleavage and IL-1β processing was assayed by western blotting. D) WT and TEV-site1 GFP-HANLRP1B were cotransfected with TEV or empty expression vector into 293T cells. Thirty-six hours post transfection cells were treated with LeTx for 4 h, then lysed and analyzed by immunoblotting with an anti-HA antibody. (TIF) [file ppat.1003452.s001.tif]

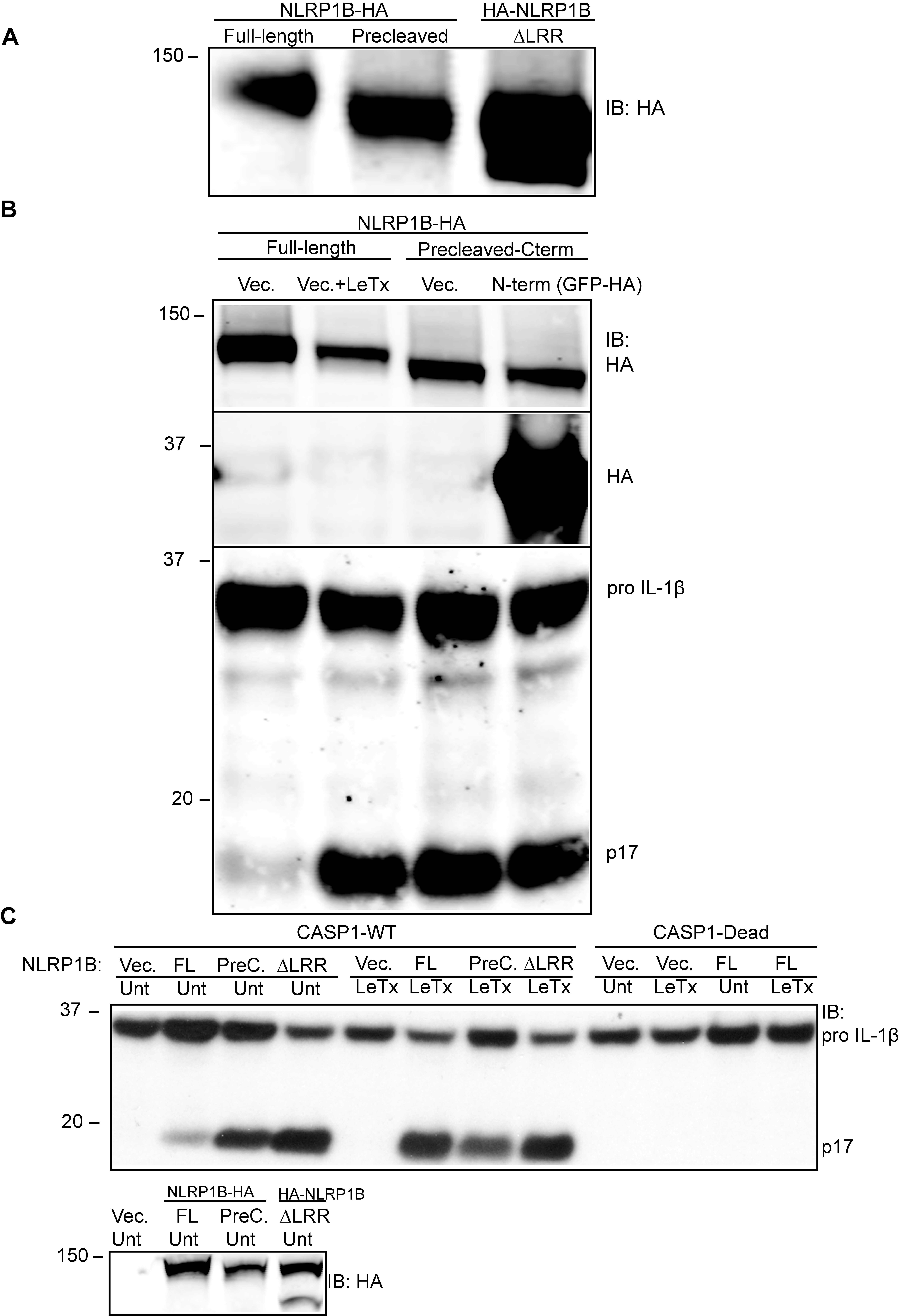

Supplement: Figure S3 — NLRP1B's N-terminal fragment has no role in inflammasome activation when expressed in trans. A) Expression of FL, precleaved and ΔLRR mutants of NLRP1B were determined by anti-HA immunoblotting. B) 293T cells were cotransfected with C-terminally HA tagged WT or precleaved NLRP1B along with Casp1 and Il1b expression constructs. The N-terminal fragment (residues 1–44) fused to GFP-HA was co-transfected with the C-terminal fragments and assayed for IL-1β 24 h post-transfection. C) Processing of IL-1β in 293T cells was determined to be dependent on NLRP1B expression and the catalytic activity of CASP1. (TIF) [file ppat.1003452.s003.tif]

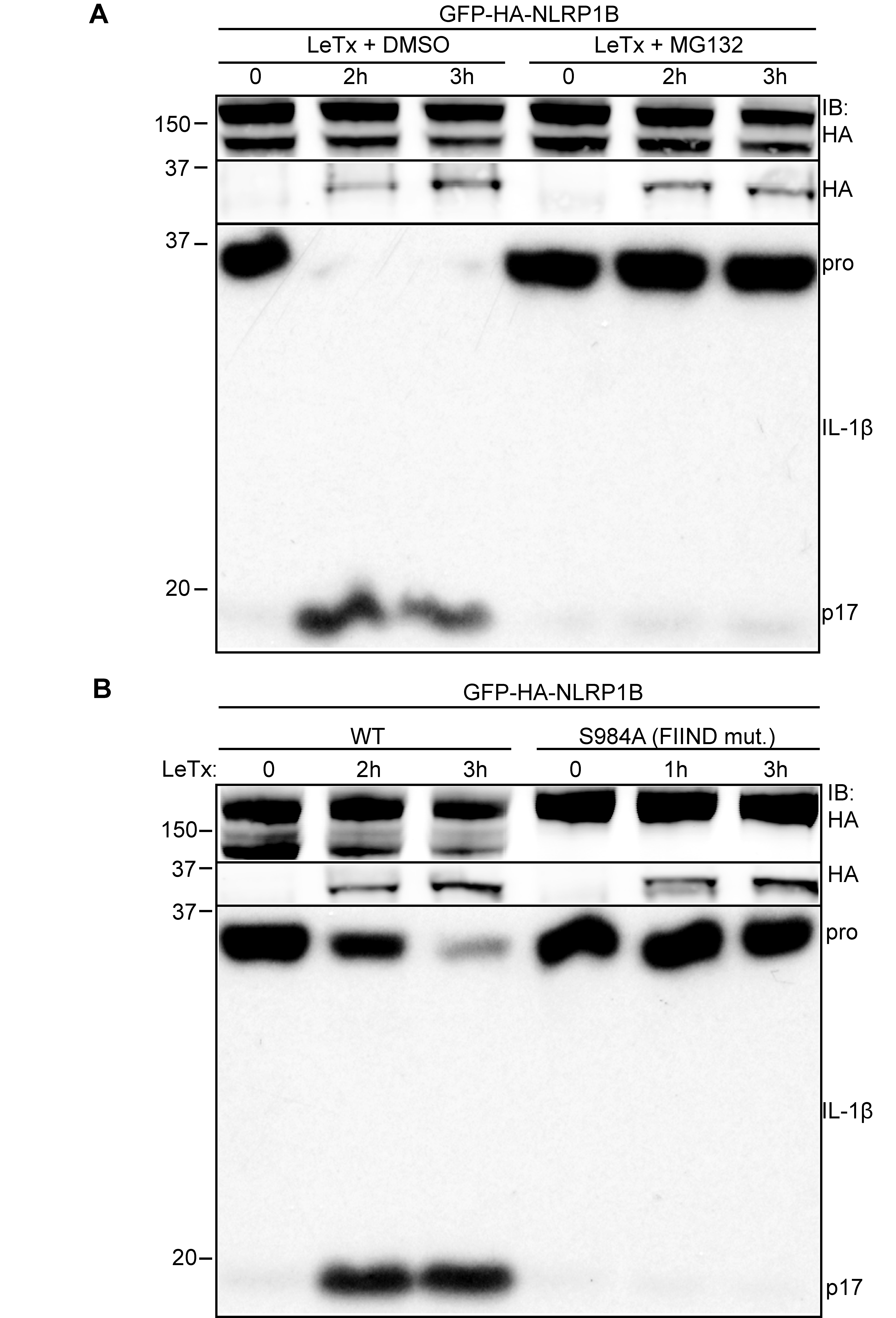

Supplement: Figure S4 — MG132 blocks NLRP1B activity and FIIND processing is required in 293T cells. A) IL-1β processing was analyzed in 293T cells expressing GFP-HA-NLRP1B, Casp1, Il1b and treated with MG132 (proteasome inhibitor) and LeTx for the indicated time points. B) The necessity of FIIND domain processing for NLRP1B activation in 293T cells was determined by measuring IL-1β processing in cells expressing GFP-HA-NLRP1B, Casp1, Il1b, after treatment with LeTx for the indicated time points. (TIF) [file ppat.1003452.s004.tif]
